# Gastroesophageal reflux in mechanically ventilated pediatric patients and its relation to ventilator-associated pneumonia

**DOI:** 10.1186/cc8134

**Published:** 2009-10-19

**Authors:** Tarek A Abdel-Gawad, Mostafa A El-Hodhod, Hanan M Ibrahim, Yousef W Michael

**Affiliations:** 1 Pediatric Department, Ain Shams Faculty of Medicine, Abbassia Ramsis St., Cairo, 11566, Egypt

## Abstract

**Introduction:**

The objective was to determine the frequency of gastroesophageal reflux (GER) in mechanically ventilated pediatric patients and its role as a risk factor for ventilator-associated pneumonia (VAP), which may be enhanced among those patients.

**Methods:**

The study is a prospective cohort study of mechanically ventilated pediatric patients in the pediatric intensive care unit (PICU) of Ain Shams University Children's Hospital. It was conducted in 24 mechanically ventilated patients (16 of them developed VAP and 8 did not, with mean age of 16.6 +/- 20.5 and 18.6 +/- 22.4 months respectively). Esophageal 24-hour pH-metry beside clinical and laboratory evaluation of their underlying problem and severity of their condition were carried out.

**Results:**

All VAP patients had GER (50% alkaline reflux, 12.5% acidic reflux and 37.5% combined reflux) compared to 75% of non-VAP ones (100% alkaline reflux). The mean total reflux time was significantly longer among VAP (50 minutes) versus non-VAP (3 minutes) patients. There was significant increase in acidic reflux parameters among non-survivors versus survivors (P < 0.001). For VAP mortality, total acidic reflux at a cut-off value of 28.6 minutes is found to be a mortality predictor with a sensitivity of 100% and a specificity of 100%.

**Conclusions:**

GER is a constant incident in mechanically ventilated pediatric patients, with alkaline reflux being more common than acidic reflux. Both acidic and alkaline refluxes were found to be associated with the development of VAP and total reflux time was found to be a reliable predictor of VAP. Moreover, acidic reflux was found to be more related to mortality than alkaline reflux.

## Introduction

Ventilator associated pneumonia (VAP) refers specifically to nosocomial pneumonia that has developed in patients who are receiving mechanical ventilation. VAP that occurs within 48 to 72 hours after tracheal intubation is usually termed early-onset pneumonia; it often results from aspiration, which complicates the intubation process [1].

One of the most common recognized risk factors for VAP is the bacterial colonization of the gastric content with subsequent gastro-esophageal reflux (GER) and aspiration into the airways [2].

In mechanically ventilated patients in the pediatric intensive care unit (PICU), the physiological environment differs substantially from that in the awake subject [3]. Low or absent lower esophageal sphincter (LES) pressure in critically ill patients may be due to many factors including drugs such as adrenergic agonists, bronchodilators and opiates used for sedation during mechanical ventilation. Also, hypotension and sepsis may contribute [4].

The acid clearance of the esophagus is a two-step process: volume clearance by esophageal peristalsis followed by chemical neutralization by swallowed salivary bicarbonate. Both of these elements are likely to be severely impaired in ventilated patients, as well as the esophageal motility being markedly impaired and salivary secretion being diminished by sleep [5]. Also supine body positioning is one of the most important predisposing factors of GER and aspiration in mechanically ventilated patients [6].

We hypothesized that GER occurs in ventilated non-pneumonic infants and children and is possibly a predisposing factor for VAP development in such patients. So the aim of the work was to determine the frequency of GER in mechanically ventilated patients and its role as a risk factor for VAP.

## Materials and methods

This prospective cohort study was conducted in the PICU of the Ain Shams University Children's Hospital. In this PICU the admission rate is 30 patients per month, with the total mortality in 2007 being 30%. The overall rate of mechanical ventilation in the same year was 70% with half of these patients developing VAP. The mortality among the VAP patients was very high (70%). Twenty-four critically ill pediatric patients admitted from March 2007 to September 2007 were chosen for the present study. All patients were mechanically ventilated and had been followed to select cases of late-onset VAP (after the first 72 hours of mechanical ventilation) by broncho-alveolar lavage (BAL) cultures at end of third, fifth and seventh day of mechanical ventilation. Moreover, if the Clinical Pulmonary Infection Score (CPIS) was found to be above 6 at any time, additional BAL culture was considered. All patients were mechanically ventilated on pressure mode Pressure-Synchronized Intermittent Mandatory Ventilation (P-SIMV) and Continuous Positive Airway Pressure with Pressure support (CPAP with PS) using non-cuffed Polyvinyl endotraceal tubes. Patients with evidence of pneumonia at the time of mechanical ventilation, patients on antacids, H2 blockers or proton pump inhibitors were excluded from the study. All patients were nutritionally supported through total parenteral nutrition during the study period and use of nasogastric tube feeding was an exclusion criterion.

The study protocol was approved by the ethical committee of the Pediatrics Department, Ain Shams Faculty of Medicine in December 2006. Informed written consent was given by the parents/caregivers of all patients. When parents/caregivers were illiterate, the consent was read to them by a hospital social worker who was not involved in the study, and the consent form was witnessed by an independent third party.

A full medical history was taken from all patients which included name, age, sex, date, and cause of PICU admission, duration of PICU stay, setting of mechanical ventilation, history of medications, type of nutrition and the fate of all patients. Full clinical examination and estimation of the angle of back rest elevation was performed for all subjects. Evaluation was performed through applying the 'pediatric risk of mortality score' (PRISM II) [7] at the time of admission.

All subjects were studied with esophageal 24 hours pH-metry using Quick Start Orion II pH monitor MMS (Medical Measurement Systems Company, Enschede, The Netherlands) using a single-use, one-way catheter. The study has been performed within the first 24 hours from starting mechanical ventilation and after hemodynamic stabilization of the patient. The catheter was calibrated before use with two solutions, one acidic (pH = 4) and the other alkaline (pH = 7). The catheter was inserted in the lower esophagus 5 cm above the gastro-esophageal sphincter (as detected by a plain film), and then the pH was recorded in 24 hours. The pH monitor recorded the reflux either acidic or alkaline through recording the pH, total reflux time, number of reflux episodes, and longest reflux [8]. The reflux was considered pathological when the total reflux time exceeds 4% of total recording time [9].

### Statistical methods

All data were tabulated. Software Package for Social Statistics version 13 (SPSS Inc., Chicago, IL, USA) was used for statistical analysis. Numerical data were expressed as mean ± standard deviation, while categorical data were expressed as number and percent. A chi squared test was used to compare two groups of categorical data e.g., sex. Unpaired t-student test was used to compare numerical parametric data e.g., age. Mann-Whitney U test was used to compare two groups of non-parametric data e.g., results of pH metry. Pearson r-test was used to correlate different parameters. Sensitivity and specificity were calculated to determine the predictive accuracy of different diagnostic test. Receiver Operating Characteristic (ROC) curves were used to illustrate the relation between sensitivity (proportion of true positive results) and specificity (proportion of false positive results). The area under the ROC curve (AUC) was determined and considered to be of good accuracy if more than 0.70. *P *value was calculated after each statistical test, and considered to be significant if less than 0.05 and highly significant if less than 0.01.

## Results

According to BAL results, patients were subdivided into two groups. Group A: This group included patients with VAP. There were 16 patients with a mean age of 16.6 ± 20.5 months. There were 12 males (75%) and 4 females (25%), and after follow up of these cases, they had been subdivided into 12 non-survivors and 4 survivors patients. Group B: This included patients who did not develop VAP and acted as a control group. There were 8 patients with a mean age of 41 ± 50.7 months. There were 6 males and 2 females.

Age was not statistically different between the VAP patients and the control patients without VAP (mean age of 16.6 ± 20.5 and 18.6 ± 22.4 months, respectively). The original diagnosis among VAP patients was 2 with encephalitis, 1 with Guillain Barre Syndrome, 1 with Werding Hoffman disease, 4 with gastro-enteritis and shock, 2 with intracranial hemorrhage, 2 with onchological problems with central nervous system (CNS) infiltrates, 2 with acute severe asthma and 2 patients with acute bronchiolitis. The original diagnosis among non-VAP patients was 1 with encephalitis, 1 with Werding Hoffman disease, 2 with atonic cerebral palsy, 2 with acute leukemia with CNS infiltrates and 2 patients with congenital cyanotic heart disease. There was no statistically significant difference between VAP and non-VAP patients as regards PRISM II score (18.7 ± 3.6 versus 17 ± 3.2, respectively). Similarly, there was no significant difference between VAP and non-VAP patients as regards the initial ventilatory settings (Respiratory rate was 48.58 ± 0.51 versus 41.50 ± 10.89; *P *> 0.05, Peak Inspiratory Pressure (PIP) was 17.00 ± 2.71 versus 17.65 ± 0.81; *P *> 0.05, positive end-expiratory pressure (PEEP) was 4.00 ± 0.00 versus 4.00 ± 0.00, PS was 12.40 ± 0.27 versus 13.10 ± 0.94; *P *> 0.05 and fraction of inspired oxygen (FiO2) was 79.8 ± 0.8% versus 75.00 ± 1.12; *P *> 0.05). On day 5, the ventilatory settings were worsened in the VAP group and started to improve in the non-VAP one (respiratory rate was 55.13 ± 0.45 versus 38.12 ± 1.13, *P *< 0.05, PIP was 20.02 ± 0.4 versus 14.12 ± 0.65, *P *< 0.05, PEEP was 6.000 ± 0.08 versus 4.000 ± 0.00, *P *< 0.05, PS was 12.402 ± 0.27 versus 8.12 ± 0.36, *P *< 0.05, FiO2 was 80.24 ± 0.9% versus 50.14 ± 3.43, *P *< 0.05)

Regarding the drug use for patients, sedative use was not significantly different between VAP (10/16) and non-VAP (6/8) patients (*P *> 0.05). Inotropes have been used with the initiation of mechanical ventilation in 2 of 8 non-VAP patients and 5 of 16 VAP patients with a non-significant difference. Additionally, 9 VAP patients needed inotropes that were started from day 4 to day 7 after development of VAP because of a deterioration in their condition. All non-survivors were on inotropes.

BAL culture results revealed that *Klebsiella *was the most common organism responsible for VAP among this group (7/16), followed by *Acinetobacter *(5/16), *Staphylococcus aureus *(2/16) and *Enterococci *(2/16)

GER was demonstrated in all patients with VAP (100%) compared with non-VAP (75%) patients. Alkaline reflux was the most frequent finding seen in both groups with no significant difference between them. Acid reflux whether isolated or combined with alkaline reflux was significantly seen in VAP (50%) compared with non-VAP (0%) patients.

Results of 24 hours pH-metry (Table 1) showed that total acid reflux time and its percentage, number of acid reflux episodes, number of long acid reflux episodes (>5 minutes), longest acid reflux time in minutes, acid reflux index and total reflux time were significantly higher among VAP compared with non-VAP patients.

**Table 1:** Comparison of pH metric results (median and range) in patients with ventilator associated pneumonia and controls

pH metric results	Patients (n = 16)	Control (n = 8)	*P*-value by MWUT
Total acid reflux time (min) (pH ≤ 4) (acid pH)	50 (1.75-393)	3 (0.7-4.4)	<0.05
Total acid reflux time (%) (pH ≤ 4%) (acid pH)	3.9 (0.12-31.3)	0.2 (0.05-2.3)	<0.05
Number of acid reflux episodes	6.5 (0.75-16.5)	1 (0.25-2.5)	<0.05
Number of long acid reflux episode >5 minutes	1.5 (0-3.7)	0 (0-0)	<0.05
Longest acid reflux time	40.9 (0.97-140)	2.6 (0.4-4.42)	<0.05
Lowest pH reached	0 (0-3.9)	1.15 (0-2.97)	>0.05
Acid reflux index	0.35 (0.02-70)	0.05 (0-0.1)	<0.05
Total alkaline time (min)	1068 (238-1185)	463 (221-847)	>0.05
Total alkaline time (%)	69.3 (27.1-89.2)	36.2 (7.9-60)	>0.05
Total reflux time (%)	83.2 (45.6-93.4)	36.3 (18-61)	<0.05

MWUT = Mann-Whitney U-test; VAP = ventilator acquired pneumonia.

The mortality of VAP patients was seen to be significantly high among acid reflux (100%) and mixed reflux (100%) patients compared with alkaline reflux (50%) patients. Overwhelmingly, sepsis and sepsis-induced multi-organ system failure was the direct cause of death in all cases.

Regarding the acid reflux parameters (Table 2) in relation to mortality outcome it was shown that total acid reflux time and its percentage, number of acid reflux episodes, number of long acid reflux episodes (>5 minutes), longest acid reflux time and acid reflux index were significantly higher among non-survivors than survivors with VAP. Lowest pH reached was significantly lower in non-survivors compared with survivors. Alkaline reflux parameters were not different between survivors and non-survivors.

**Table 2:** Comparison of pH metric results and total reflux time (median and range) in survivors and non survivors among cases of ventilator associated pneumonia

Variables	Survivors (n = 4)	Non survivors (n = 12)	*P*-value by MWUT
Total acid reflux time (min) (pH ≤ 4)	0 (0-0)	183 (29-425.5)	<0.01
Total acid reflux time (%) (pH ≤ 4%)	0 (0-0)	14.4 (2.1-34)	<0.01
Number of acid reflux episodes	0 (0-0)	9.5 (6-18)	<0.01
Number of long acid reflux episode >5 mins	0 (0-0)	2.5 (1-4)	<0.05
Longest acid reflux time	0 (0-0)	91.8 (26.2-144.9)	<0.01
Lowest pH reached	5 (4.7-5.3)	0 (0-0)	<0.01
Acid reflux index	0 (0-0)	0.55 (0.3-0.7)	<0.01
Total alkaline time (min)	636 (65.8-1206)	1068 (755-1122)	>0.05
Total alkaline time (%)	53 (21.9-85.5)	69.3 (42.7-90)	>0.05
Total reflux time (%)	53 (21.9-85.5)	83 (80.1-96)	>0.05

MWUT = Mann-Whitney U-test.

ROC curves of predictability of VAP and mortality showed that a total reflux time of 74.65 minutes per day has 75% sensitivity and 100% specificity for VAP development, while acidic reflux time of 28.6 minutes per day has 100% sensitivity and specificity for mortality prediction among VAP patients.

## Discussion

In the current study we found that GER occurred in up to 91.6% of all mechanically ventilated critically ill patients of either VAP or non-VAP patients. Many previous studies reported that the incidence of GER is increased in critically ill mechanically ventilated patients with the acidic reflux reaching up to 80% and bile reflux (duodenogastroesophageal reflux (DGER)) reaching up to 60% [3,10]. Another study found that the GER reached up to 74% in patients with nasogastric tube (NGT) which increased to 81% in supine position [11]. The patients are defined to have abnormal esophageal bile reflux (DGER) if the fraction of the time that the esophageal mucosa is exposed to alkaline refluxate exceeds 4% of the total study time and pathological acidic reflux if the fraction of the time that the esophageal mucosa is exposed to a refluxate with pH less than 4 exceeds 4% of the total recording time [12]. Previous studies demonstrated that sedatives [3] and adrenergic drugs [13] do increase reflux. In the current study, a great proportion of the studied patients were on those suspected medications; however, the frequency of use was not different between VAP and non-VAP groups.

In the present study, there was a significant increase in GER parameters including total acidic time, number of reflux episodes, number of long reflux episodes longer than five minute, longest reflux time, reflux index and total reflux time in VAP than non-VAP patients. Alkaline reflux was more common than acidic one, and there was no statistically significant difference in alkaline pH parameters between VAP and non-VAP patients. The use of acid suppressive medications were reported to increase frequency of non acid reflux [14]; however, in the present study, patients using these medications were excluded. The demonstrated alkaline reflux can be explained by duodenogastric reflux. Duodenogastric reflux is the retrograde flow of duodenal contents into the stomach that then mix with acid and pepsin. These agents can reflux into the esophagus (*ie*, DGER) and cause gastro-esophageal reflux disease [15].

The mortality rate in our study among VAP patients was 75%, but in non-VAP patients was 25%. The difference was statistically significant. In general this high mortality rate can be attributed to the highly pathogenic organisms that have been isolated in cultures as well as the pulmonary devitalizing effect of severe acid reflux. This was in agreement with other work [16,17] that reported high mortality rate of VAP patients.

In the current study, all acidic parameters of pH metric results were significantly higher among non-survivors indicating a strong relation between acidic reflux and high mortality rate in VAP patients. Acid and pepsin are the major factors responsible for symptoms and esophageal mucosal damage in gastro-esophageal reflux disease. Several investigators, however, have shown that the association between frequency of symptoms and severity of mucosal lesions on one hand, and the amount of the esophageal acid exposure on the other one, is not as straightforward as one might expect. The occurrence of symptoms is influenced by mucosal sensitivity, which is only partly dependent on increased esophageal acid exposure [18].

In view of similar base line PRISM results and other circumstantial risk factors between VAP and non-VAP groups, the significant association between acidic reflux and mortality in VAP group points to its detrimental effects in this group of patients.

## Conclusions

In conclusion, GER is a permanent incident in mechanically ventilated pediatric patients who were not on antacid therapy. Alkaline reflux is found to be more common than acidic reflux. Both acidic and alkaline refluxes were found to be associated with the development of VAP. Total reflux time was found to be a reliable predictor of developing VAP in these patients. Nevertheless, acidic reflux was found to be related more to high mortality among them.

## Key messages

• GER is almost constant in ventilated children and is greatly linked to development of VAP.

• Alkaline reflux is more common than acidic reflux.

• Acidic reflux is more linked to mortality in such a group of patients.

## Abbreviations

AUC: area under ROC curve; BAL: bronchoalveolar lavage; CPIS: Clinical Pulmonary Infection Score; CNS: central nervous system; DGER: dudenogastroesophageal reflux; GER: gastroesophageal reflux; LES: lower esophageal sphincter; PEEP: positive end-expiratory pressure; PICU: pediatric intensive care unit; PRISM: Pediatric Risk of Mortality Score; ROC: receiver operating characteristic; VAP: ventilator-associated pneumonia.

## Competing interests

The authors declare that they have no competing interests.

## Authors' contributions

TAA established the idea and revised the work as well as supervision of PICU management of the patients. MAE performed the pH metry with its preparation and prescribed the suitable treatment as well as writing and preparation of the manuscript. HMI performed the clinical recruitment and evaluation of patients, PICU management of the patients, statistical analysis of the results and discussion of the work. YWM performed the collection of literature relevant to the subject and tabulation of data.

## References

[B1] Chastre J Fagon JY Diagnosis of ventilator-associated pneumonia N Engl J Med 2007 356 1469 10.1056/NEJMc076017 17409331

[B2] du Moulin GC Paterson DG Hedley-Whyte J Lisbon A Aspiration of gastric bacteria in antacid-treated patients: a frequent cause of postoperative colonization of the airway Lancet 1982 1 242 245 10.1016/S0140-6736(82)90974-6 6120273

[B3] Nind G Chen WH Protheroe R Iwakiri K Fraser R Young R Chapman M Nguyen N Sifrim D Rigda R Holloway RH Mechanisms of gastro-esophageal reflux in critically ill mechanically ventilated patients Gastroenterology 2005 128 600 606 10.1053/j.gastro.2004.12.034 15765395

[B4] Fan YP Chakder S Gao F Rattan S Inducible and neuronal nitric oxide synthase involvement in lipopolysaccharide-induced sphincteric dysfunction Am J Physiol Gastrointest Liver Physiol 2001 280 G32 42 11123195 10.1152/ajpgi.2001.280.1.G32

[B5] Kölbel CB Rippel K Klar H Singer MV van Ackern K Fiedler F Esophageal motility disorders in critically ill patients: a 24-hour manometric study Intensive Care Med 2000 26 1421 1427 10.1007/s001340000630 11126251

[B6] Torres A Serra-Batlles J Ros E Piera C Puig de la Bellacasa J Cobos A Lomeña F Rodríguez-Roisin R Pulmonary aspiration of gastric contents in patients receiving mechanical ventilation the effect of body position Ann Intern Med 1992 116 540 543 1543307 10.7326/0003-4819-116-7-540

[B7] Pollack MM Ruttimann UE Getson PR Pediatric risk of mortality (PRISM) score Crit Care Med 1988 16 1110 1116 3048900 10.1097/00003246-198811000-00006

[B8] Cavataio F Guandalini S Guandalini S Gastroesophageal reflux Essential Pediatric Gastroenterology and Nutrition 2005 New York, NY: McGraw-Hill 157 173

[B9] Wilmer A Tack J Frans E Dits H Vanderschueren S Gevers A Bobbaers H Duodenogastroesophageal reflux and esophageal mucosal injury in mechanically ventilated patients Gastroenterology 1999 116 1293 1299 10.1016/S0016-5085(99)70492-0 10348811

[B10] Xin Y Dai N Zhao L Wang JG Si JM The effect of famotidine on gastro-esophageal and duodenogastro- esophageal refluxes in critically ill Patients World J Gastroenterol 2003 9 356 358 12532466 10.3748/wjg.v9.i2.356 PMC4611346

[B11] Ibáñez J Peñafiel A Marsé P Jordá R Raurich JM Mata F Incidence of gastroesophageal reflux and aspiration in mechanically ventilated patients using small-bore nasogastric tubes JPEN J Parenter Enteral Nutr 2000 24 103 106 10.1177/0148607100024002103 10772190

[B12] Richter JE Bradley LA DeMeester TR Wu WC Normal 24-hr ambulatory esophageal pH values. Influence of study center, pH electrode, age and gender Dig Dis Sci 1992 37 849 856 10.1007/BF01300382 1587189

[B13] Crowell MD Zayat EN Lacy BE Schettler-Duncan A Liu MC The effects of an inhaled beta (2)-adrenergic agonist on lower esophageal function: a dose-response study Chest 2001 120 1184 1189 10.1378/chest.120.4.1184 11591558

[B14] Orr WC Craddock A Goodrich S Acidic and non-acidic reflux during sleep under conditions of powerful acid suppression Chest 2007 131 460 465 10.1378/chest.06-1312 17296648

[B15] Hak NG Mostafa M Salah T El-Hemaly M Haleem M Abd El-Raouf A Hamdy E Acid and bile reflux in erosive reflux disease, non-erosive reflux disease and Barrett's esophagus Hepatogastroenterology 2008 55 442 447 18613384

[B16] Alp E Güven M Yildiz O Aygen B Voss A Doganay M Incidence, risk factors and mortality of nosocomial pneumonia in intensive care units: a prospective study Ann Clin Microbiol Antimicrob 2004 3 1 17 10.1186/1476-0711-3-1 15369593 PMC521500

[B17] Srinivasan R Asselin J Gildengorin G Wiener-Kronish Flori HR A prospective study of ventilator-associated pneumonia in children Pediatrics 2009 123 1108 1115 10.1542/peds.2008-1211 19336369

[B18] Trimble KC Pryde A Heading RC Lowered esophageal sensory thresholds in patients with symptomatic but not excess gastro-esophageal reflux: evidence for a spectrum of visceral sensitivity in GERD Gut 1995 37 7 12 10.1136/gut.37.1.7 7672684 PMC1382759

